# Development of a Tool for High‐Efficiency, Markerless and Iterative Genome Editing in *Shouchella clausii*

**DOI:** 10.1111/1751-7915.70287

**Published:** 2026-02-09

**Authors:** Claudia Cappella, Carsten Jers, Lorenzo Ninivaggi, Maurizio Bettiga, Ivan Mijakovic, Gennaro Agrimi, Pasquale Scarcia

**Affiliations:** ^1^ Department of Biosciences, Biotechnologies and Environment University of Bari “Aldo Moro” Bari Italy; ^2^ Novo Nordisk Foundation Center for Biosustainability Technical University of Denmark Kongens Lyngby Denmark; ^3^ Italbiotec Srl Società Benefit Milan Italy; ^4^ Systems and Synthetic Biology Chalmers University of Technology Gothenburg Sweden

**Keywords:** *Bacillus clausii*, electroporation, gram‐positive bacteria, homologous recombination, markerless genome editing, *Shouchella clausii*, temperature‐sensitive replication

## Abstract

*Shouchella clausii* is a spore‐forming, Gram‐positive bacterium with intrinsic antibiotic resistance and promising potential in biotherapeutics, industrial biotechnology and environmental applications. Its genetic intractability, due to a rigid cell wall and lack of natural competence, has limited its development as a microbial chassis. To facilitate its genetic transformation, a hyperosmotic electroporation protocol was optimised using cell wall weakening agents, achieving efficiencies comparable to other recalcitrant bacilli. A comprehensive and reusable genetic tool was developed centred on a temperature‐sensitive *E. coli–S. clausii* shuttle vector (pM4B522) specifically engineered for compatibility with Golden Gate assembly. The plasmid backbone includes a spectinomycin resistance marker and an integrated red fluorescent protein reporter for transformants selection. A removable AmilCP chromoprotein cassette streamlines the assembly process by enabling blue/white screening in *E. coli* . This plasmid, employing a two‐step pop‐in/pop‐out integration strategy, has demonstrated high versatility for genome editing in both *S. clausii* and *Bacillus subtilis* as evidenced by its successful use in multiple cases: (i) sequential, markerless deletions of the non‐essential catabolic genes *xylA* and *lacA* in *S. clausii* DSM 8716, with a success rate exceeding 60%; (ii) replacement of the *lacA* coding sequence with a *gfp* coding sequence, resulting in fluorescence induction in lactose‐supplemented medium; (iii) introduction of a single‐base substitution generating a premature stop codon in *lacA*, showcasing scar‐free point mutagenesis; and (iv) transfer of the system to *B. subtilis* 168, highlighting its broader applicability across Gram‐positive bacteria. Given the precision and scarless nature of these genetic modifications, this system holds strong potential for the development of next‐generation probiotics and synthetic biology applications.

## Graphical abstract

*Shouchella clausii*, a spore‐forming Gram‐positive bacterium with a long‐standing use as probiotic, has long been considered genetically intractable. This study introduces a high‐efficiency electroporation protocol and a versatile Golden Gate‐compatible shuttle vector (pM4B522), enabling precise, scarless and iterative genome editing in both *S. clausii* and *Bacillus subtilis*, and unlocking new possibilities for applications in biotherapeutics, industrial biotechnology and environmental science.

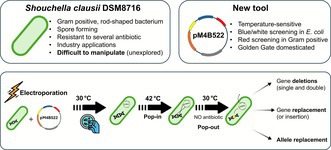

## 1 Introduction

*Shouchella clausii* (formerly *Bacillus clausii* ) (Joshi et al. 2021) is a spore‐forming, alkaliphilic, Gram‐positive bacterium that has a long history of safe use, as well as a growing number of applications in the fields of probiotics, industrial and environmental microbiology (Joshi et al. 2021; Elshaghabee et al. 2017; Nielsen et al. 1995). Beyond its probiotic role, *S. clausii* has been investigated for non‐clinical uses: *B. clausii* strain T fully degrades (mineralizes) cephalosporin antibiotics (cefuroxime, cefotaxime, cefpirome) in wastewater (Kong et al. 2019); isolate I‐52 secretes an alkaline protease that, after 72 h, retains more than 75% of its activity in 5% SDS and about 110% of its initial activity in 10% H_2_O_2_, a property valued in the detergent industry (Joo et al. 2003); spores and their glycopolypeptide biosurfactant have been incorporated into silica sol–gel coatings to inhibit biofilm‐driven corrosion of carbon steel (Purwasena et al. 2023); and the rhizobacterial strain B8 promotes biomass accumulation in *Brassica napus* and *Medicago sativa* , highlighting its potential as a biofertilizer (Oulebsir‐Mohandkaci et al. 2021). Since *S. clausii* is already approved for human use, its application in industrial, agricultural, or waste‐remediation contexts faces fewer regulatory and practical hurdles.

The species has been commercialised worldwide for over 60 years in high‐spore‐density, over‐the‐counter spore formulations such as Enterogermina (Italy; a four‐strain cocktail, SIN/O‐C/T/N‐R, 2–4 billion spores per vial) and Erceflora (South‐East Asia and Latin America; 2 billion spores per vial). Furthermore, clinical randomised trials showed that strain 088AE lowers both the incidence and severity of antibiotic‐associated diarrhoea when co‐administered with broad‐spectrum antibacterials (Maity and Gupta 2021), and additional studies report benefits in paediatric upper respiratory tract infections and persistent diarrhoea (Sudha et al. 2019) (Madempudi et al. 2022) (Dang et al. 2024). Crucially, comparative genomics has identified chromosomally encoded resistance déterminants, including *erm(34)*, *aadD2* and the β‐lactamase *bcl‐1*, in DSM 8716 and commercial strains; these loci are not flanked by insertion sequences or other mobile elements, no conjugative plasmids have been detected, and no study to date has demonstrated horizontal transfer in vivo (Bozdogan et al. 2003; Girlich et al. 2007). A 2022 narrative review, covering over thirty years of clinical application, reported no documented cases of in vivo horizontal gene transfer (Acosta‐Rodríguez‐Bueno et al. 2022). This evidence reframes *S. clausii* 's intrinsic antibiotic resistance profile from a regulatory liability to a clinical advantage, thereby supporting its use as an antibiotic‐compatible live biotherapeutic capable of preserving or restoring the microbiota during antimicrobial treatment.

*S. clausii* offers a unique combination of a long‐standing history of human consumption, Qualified Presumption of Safety (QPS) status and compatibility with antibiotics. These attributes position it as a lower‐risk chassis for therapeutic development (Acosta‐Rodríguez‐Bueno et al. 2022) and a particularly promising candidate for next‐generation probiotics (NGPs) and live biotherapeutic products (LBPs) (Chang et al. 2019). To develop these applications, precise genomic modifications of *S. clausii* are essential.

To our knowledge, no stably replicating plasmid or reproducible transformation workflow has yet been established for *S. clausii* . This process is complicated by the presence of a thick, highly cross‐linked peptidoglycan cell wall and the absence of key proteins required for natural transformation in Gram‐positive bacteria (Ahmed et al. 2022; Kovács et al. 2009).

In this work, we overcame key obstacles to genetic manipulation of *S. clausii* DSM 8716 by (i) optimising a high‐osmolarity electroporation method to reduce the impact of spore wall resistance, and (ii) designing a modular *E. coli* – *S. clausii* shuttle vector. This vector serves as a flexible, reusable tool for scarless gene engineering including deletions, insertions and point mutations. The resulting system enables transformation efficiencies suitable for standard molecular biology applications and supports stable recombinant gene expression.

## 2 Materials and Methods

### 2.1 Bacterial Strains and Growth Conditions

For routine cloning and plasmid propagation, *E. coli* DH5α was cultivated in Luria‐Bertani (LB) medium at 37°C, either in liquid culture with shaking or on LB agar plates. When appropriate, spectinomycin was added at a final concentration of 50 μg mL^−1^ for selection.

*S. clausii* DSM 8716, *B. subtilis* 168 and derived strains were propagated in LB medium at 30°C, 37°C or 42°C as required. Spectinomycin was added at a final concentration of 100 μg mL^−1^ when needed.

### 2.2 Antibiotic Susceptibility Assays

Antibiotic susceptibility of *S. clausii* DSM 8716 was assessed by culturing the strain in both liquid and solid LB media supplemented with various antibiotics at concentrations in the range of 1–100 μg mL^−1^. For liquid assays, cells were inoculated into LB broth containing the desired antibiotic and incubated at 37°C for 24 h with shaking; growth was monitored by measuring optical density at 600 nm (OD₆₀₀). For solid media assays, overnight cultures were plated onto LB agar containing the appropriate antibiotic and incubated at 37°C for 48 h and growth was determined by visual inspection of colony formation.

### 2.3 Molecular Cloning Procedures

Genomic DNA from *S. clausii* DSM 8716 and *B. subtilis* 168 was extracted using the DNeasy UltraClean Microbial Kit (Qiagen). Plasmid DNA was routinely isolated from *E. coli* DH5α cultures using E.Z.N.A. plasmid DNA Mini Kit I (omega BIO‐TEK), following the manufacturer's protocols.

For vector constructions, DNA fragments were amplified using the primers listed in Table S1 and relevant template DNA. PCR amplifications were performed using PrimeSTAR GXL DNA Polymerase (Takara Bio); the sequences of all homology arms are provided in Table S2.

Overlap extension PCR was performed as described by Shevchuk (2004). When required, PCR products were purified using E.Z.N.A. Cycle Pure Kit (omega BIO‐TEK) and quantified using Nanodrop 1000 Spectrophotometer (Thermo Scientific).

Relevant DNA fragments were assembled using either Gibson Assembly (NEBuilder HiFi DNA Assembly, New England Biolabs) or by Golden Gate Assembly (BsaI HiFi—New England Biolabs) and T4 DNA ligase (Thermo Scientific), following the manufacturer's protocols.

Plasmid DNA constructs were introduced into *E. coli* DH5α via chemical transformation using the Inoue method for preparing supercompetent cells (Green and Sambrook 2020). Transformed cells were then plated on LB agar containing spectinomycin and incubated overnight at 37°C. Transformants were verified by colony PCR or plasmid extraction, followed by restriction analysis and Sanger sequencing (service provided by Eurofins Genomics).

### 2.4 Gram Positive Bacteria Transformation

Transformation of *S. clausii* DSM 8716 was performed by electroporation, following a modified protocol based on Zhang et al. (2015). Briefly, to prepare competent cells, overnight cultures were diluted 1:16 into fresh LBSP medium consisting of LB supplemented with 0.5 M sorbitol, 0.05 M KH_2_PO_4_ and 0.05 M K_2_HPO_4_ (pH 7.2) and grown to an OD_600_ between 0.7 and 0.8. To enhance cell wall permeability, cultures were treated with cell wall‐weakening agents (0.5% threonine, 0.05% Tween 80 and 0.6% glycine) for 1.5 h at 37°C. Cells were harvested by centrifugation at 4°C and washed four times with ice‐cold electroporation buffer (0.33 M trehalose, 0.5 M sorbitol, 0.5 M mannitol, 0.5 mM MgCl_2_, 0.5 mM K_2_HPO_4_ and 0.5 mM KH₂PO₄; pH 7.2). The cell pellet was resuspended in the same buffer at a final volume equal to 1/60 of the initial culture volume, then divided into 50 μL aliquots. Electroporation was performed with a Gene Pulser II electroporator (Bio‐Rad) in 0.1 cm gap cuvettes at 2.2 kV, 25 μF and 200 Ω, using 60 ng of plasmid DNA or 100 ng of linear DNA per aliquot. After electroporation, cells were allowed to recover in LBMS, defined as LB supplemented with 0.5 M sorbitol and 0.38 M mannitol, at 37°C for 5 h, then plated on selective LB agar containing spectinomycin. Plates were incubated at 37°C for 36/48 h to select transformants.

Transformation of *B. subtilis* 168 was carried out using the natural competence protocol (Yasbin et al. 1975). Competent cells were incubated with 100 ng of plasmid DNA for 1.5 h at 30°C and then plated on selective LB agar.

Following transformation of *B. subtilis* and *S. clausii* , colony PCR screening was performed using a protocol adapted from the iGEM Team Technion Israel (http://2015.igem.org/Team:Technion_Israel), with substantial modifications as described below. Individual colonies of *B. subtilis* or *S. clausii* were picked from agar plates and resuspended in 10 μL of 10 mM Tris–HCl buffer pH 8.0. The suspension was incubated on ice for 10 min, then subjected to three heating cycles (two minutes at 700 W in a microwave followed by one minute at −20°C). After the final microwave step, samples were incubated on ice for an additional 15 min. Two microliters of the resulting lysate were used directly as PCR template. PCR reactions included an initial denaturation step at 95°C for 10 min to inactivate DNases released during cell lysis.

### 2.5 Construction of the lacA Deletion Cassette

The *spec* ^*r*^ coding sequence was PCR‐amplified from *B. subtilis* IHA01 (Härtl et al. 2001) genomic DNA using primers 757 (forward) and 758 (reverse; sequences in Table S1). To maintain compatibility with our cloning workflow, the amplicon was cloned into the shuttle vector pDR242a (Koo et al. 2017) by restriction‐ligation using EcoRI and XhoI (FastDigest, Thermo Fisher), generating plasmid pM4B372, which served as the PCR template for subsequent *spec* ^*r*^ amplifications.

**FIGURE 1:**
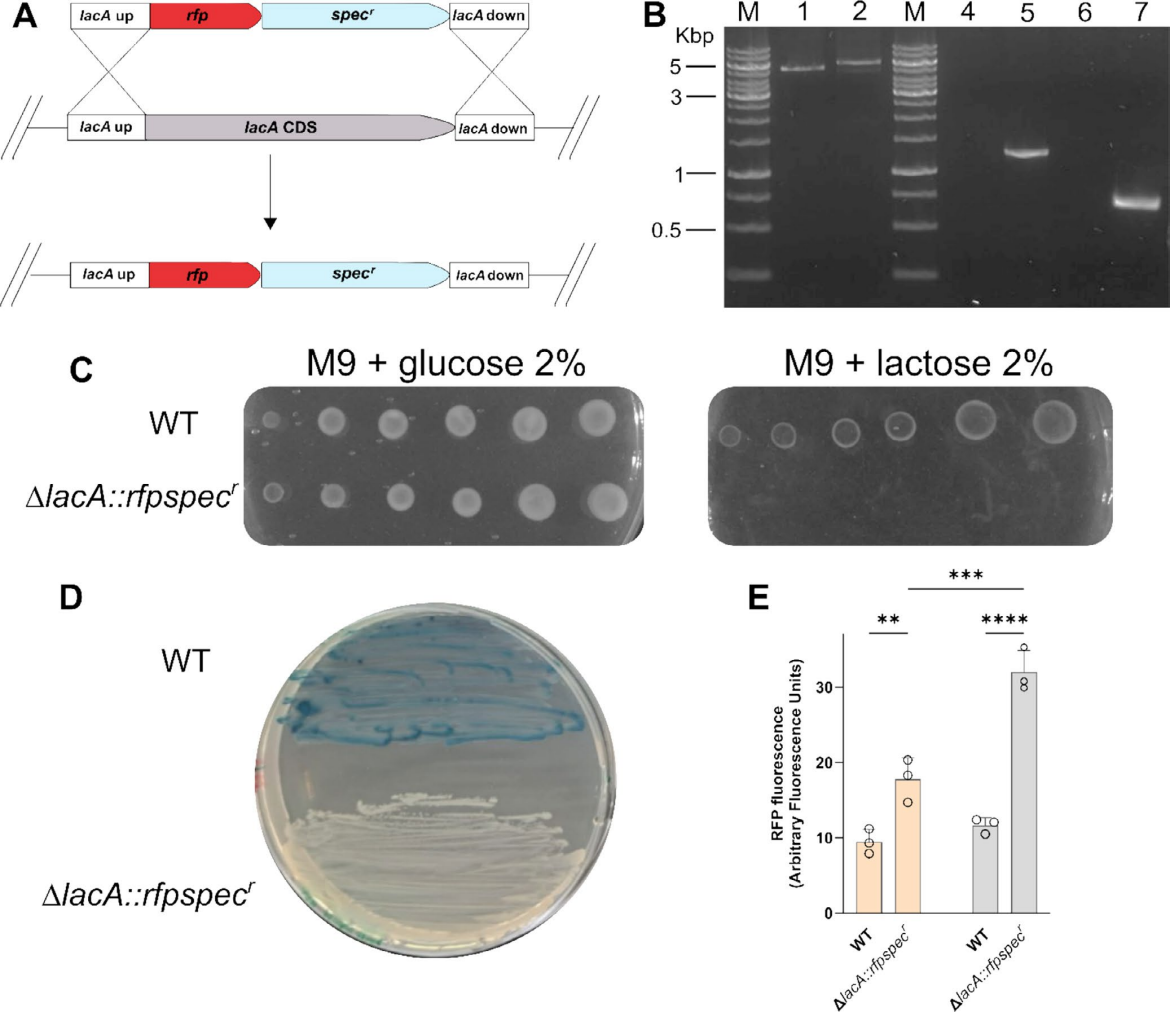
Replacement of *lacA* gene by homologous recombination. (A) Strategy for targeted replacement of the *lacA* gene in *S. clausii* DSM 8716 by homologous recombination. (B) Agarose gel analysis of colony PCR confirming cassette integration in *S. clausii* DSM 8716. Lanes M: DNA ladder (GeneRuler DNA ladder mix; Thermo Fisher); lanes 1–2: WT and ∆*lacA::rfpspec* ^*r*^ amplified with external primers (5.2 kb amplicon in the WT and 5.6 kb in recombinant clone); lanes 4–5–6‐7: WT and ∆*lacA::Rfpspec* ^*r*^ with internal *spec* ^*r*^ (1.3 kb) and *rfp* (0.7 kb) primers, respectively. C. Carbon source utilisation assay. D. X‐gal plate assay. E. RFP expression Δ*lacA::rfpspec* ^*r*^ grown in LB or LB supplemented with lactose. Fluorescence intensity of RFP expressed in Δ*lacA::rfpspec* ^*r*^ cells grown in LB (Light orange bars) and in LB supplemented with lactose (Light grey bars). Bars represent mean ± SD (*n* = 3) of fluorescence. Statistical analysis was performed using one‐way ANOVA with Tukey's post‐test (**p < 0.01; ***p < 0.001; ****p < 0.0001).

For the *lacA* integration cassette, 1.1 kb homology arms flanking *lacA* (Wu et al. 2019) were amplified from *S. clausii* DSM 8716 genomic DNA using primers 807/50 for the upstream arm and 809/810 for the downstream arm (Table S2). The *rfp* reporter was amplified from plasmid pBs1C‐RFP (Radeck et al. 2013) with primers 51/52, and the *spec* ^*r*^ marker was amplified from pM4B372 with primers 716/717. These four PCR products were assembled by overlap extension PCR (Shevchuk 2004) using nested primers 805/806 to generate a linear *lacA* replacement cassette suitable for integration in *S. clausii* (Figure 1A).

The linear *lacA* replacement cassette was introduced into *S. clausii* DSM 8716 by electroporation as described in Section 2.4. Transformants were selected on LB agar containing spectinomycin. Colonies were screened by colony PCR using an external primer pair flanking the *lacA* region—803 (forward) and 804 (reverse).

### 2.6 Construction of the Plasmids for Markerless Genome Editing (General Workflow)

All plasmids used for genome editing were constructed using a newly developed temperature‐sensitive *E. coli–S. clausii* shuttle vector, pM4B522, which was designed in this study (Supporting Information). This backbone was engineered to support one‐pot Golden Gate assembly and facilitate pop‐in/pop‐out genome editing. The plasmid includes a spectinomycin resistance gene (Härtl et al. 2001) and an RFP reporter module (Radeck et al. 2013). It also features an AmilCP chromogenic drop‐out marker (Vidal et al. 2023) flanked by inward‐facing BsaI/BsmBI Type IIS restriction sites, enabling seamless insertion of expression cassettes without leaving scars. The empty pM4B522 backbone was assembled by Gibson Assembly from two synthetic double‐stranded synthetic DNA fragments (Twist Bioscience) designed with two short overlaps to adjacent modules. The assembled plasmid was then transformed into *E. coli* for propagation. Construct identity was verified by diagnostic restriction digestion and Sanger sequencing across both junctions using primers 53 and 54 (Table S1). The fully annotated sequence is available in the Supporting Information and on the NCBI database (accession number PX529440) as a GenBank file.

The construction of the pM4B522‐based plasmids for genome editing is reported in detail in the Supporting Information. Each pM4B522‐based editing plasmid contained, from 5′ to 3′, a left homology arm between 0.8 and 1.2 kb, the segment encoding the edit, and a right homology arm between 0.8 and 1.2 kb. The central segment differed by application: (i) for deletions, the two arms were directly contiguous to excise the target region; (ii) for replacement insertions, a DNA insert (e.g., *gfp*) was placed between the arms; (iii) for allele replacement, the single‐nucleotide change was encoded in the complementary 4‐nt BsaI overhangs at the junction between the homology arms. Complete per‐edit documentation is provided in Supporting Information: Section 1, including: plasmid names, primers used to amplify fragments for Golden Gate assembly, diagnostic colony PCR primer pairs and Sanger sequencing primers. All fragments destined for insertion were amplified with primers bearing BsaI recognition sites and the appropriate four‐base overhangs.

Fragments were assembled into the backbone by one‐pot Golden Gate using BsaI‐HFv2 (New England Bioscience) together with T4 DNA ligase (New England Bioscience).

Assemblies were transformed into *E*. *coli*. Transformants carrying plasmids in which the AmilCP drop‐out cassette had been replaced appeared white (colonies with plasmids retaining AmilCP are blue/purple), enabling immediate visual screening. Candidate plasmids were screened by diagnostic restriction digests with EcoRI. The Golden Gate insert region was then verified by Sanger sequencing.

### 2.7 Gene Editing With pM4B522 and Its Derivatives

Genome editing was performed by introducing pM4B522‐derived plasmids into *S. clausii* by electroporation, followed by the pop‐in/pop‐out workflow.

Electrotransformed cells were recovered and plated on LB agar containing spectinomycin and incubated for 48 h at 30°C, a permissive temperature that allows replication of the temperature‐sensitive plasmid; resulting colonies were red and spectinomycin‐resistant due to plasmid‐borne RFP and the *spec* ^*r*^ marker.

To obtain single‐crossover integrants (pop‐in), red colonies were re‐streaked on fresh spectinomycin plates and incubated overnight at 42°C, a non‐permissive temperature at which the temperature‐sensitive origin does not support extrachromosomal replication; under these conditions, only chromosomal integrants retain spectinomycin resistance and RFP.

For resolution (pop‐out), confirmed integrants were inoculated into antibiotic‐free LB and grown overnight at 30°C to allow a second homologous recombination that excises the plasmid backbone. Cultures were then streaked on LB without antibiotic and incubated overnight at 37°C to eliminate residual plasmid‐bearing cells. Loss of the plasmid module was identified by the disappearance of red colour and by spectinomycin sensitivity.

Because the second crossover can resolve at either homology arm, the outcomes are expected to be approximately 50:50 between restoration of the wild‐type allele and retention of the edited allele. Putative genome‐modified clones were distinguished from wild type by diagnostic PCR using external (locus‐flanking) and edit‐specific primers, and were confirmed by Sanger sequencing. Details of the primer sets used for each edit (diagnostic PCR and sequencing) are provided in Supporting Information: Section 1.

### 2.8 Phenotypic Validation of Recombinant Strains

To confirm the expected phenotypes of recombinant strains, functional assays were performed.

For assessment of β‐galactosidase activity, colonies were plated on LB agar supplemented with 200 μg mL^−1^ X‐gal (5‐bromo‐4‐chloro‐3‐indolyl‐β‐D‐galactopyranoside) and incubated at 37°C overnight. Colony colour (blue or white) was assessed to distinguish wild‐type and mutant phenotypes, respectively.

Growth on various carbon sources was assessed using drop tests on M9 minimal agar plates supplemented with 2% glucose, lactose, or xylose, as appropriate. Plates were incubated overnight at 37°C.

For fluorescence reporter assays, recombinant strains were grown either in LB medium alone or in LB supplemented with 0.5% lactose for 24 h at 37°C, to assess induction of the *lacA* promoter. Cultures were harvested, washed and resuspended in sterile water to an OD_600_ of 1. Fluorescence was measured using a PerkinElmer Luminescence Spectrometer LS 50B, with excitation/emission wavelengths of 558/583 nm for RFP and 488/510 nm for GFP. Results are expressed as mean ± standard deviation (SD) from three independent experiments; statistical significance was determined using one‐way ANOVA with Tukey's post‐test, with *p* < 0.05 considered significant.

α‐Amylase activity was assessed on starch agar plates using iodine staining. Bacterial colonies were grown on LB agar supplemented with 1% (w/v) soluble starch and incubated at 37°C for 24 h. Plates were exposed to iodine vapours released from iodine crystals. The formation of clear halos around the colonies indicated starch degradation, confirming α‐amylase activity (Guérout‐Fleury et al. 1996). Plates were photographed both before and after iodine exposure to document the results.

### 2.9 BLAST Search and Phylogenetic Analysis

*S. clausii* genomes available at NCBI were screened with the sequences of *B. subtilis* 168 XylA (BSU17600) and GanA (BSU34130) proteins using TBLASTN. The amino acid sequences were aligned with ClustalW and visualised with JALVIEW v2.11.4.1. The phylogenetic tree was generated from the ClustalW multiple‐sequence alignment using the Maximum Likelihood method in MEGA11 and included the putative LacA sequences from *B. subtilis* and *S. clausii* strains.

## 3 Results

### 3.1 Transformation of *S. clausii* DSM 8716

To enable genetic engineering of *S. clausii* DSM 8716, we developed an efficient transformation protocol employing antibiotic‐based selection. *S. clausii* DSM 8716 displayed resistance to several antibiotics commonly used as selection markers, including erythromycin, chloramphenicol and kanamycin but not spectinomycin (Table S3), which was therefore chosen as the antibiotic marker for the transformation experiments.

To explore possible transformation strategies, the deletion of two non‐essential genes, *xylA*, encoding xylose isomerase and *lacA*, encoding β‐galactosidase, was carried out using homologous recombination cassettes (Koo et al. 2017). These genes were selected due to the easily scorable phenotypes resulting from their loss in *Bacillus subtilis* (Gärtner et al. 1988; Daniel et al. 1997).

BLAST searches identified orthologues of XylA and GanA (LacA) of *B. subtilis* in *S. clausii* DSM 8716. For XylA, a single ortholog (AST95085.1; 440 aa) was identified, showing 73.2% amino acidic identity to the *B. subtilis* enzyme (Figure S1).

For β‐galactosidase, tBLASTP with the *B. subtilis* GanA protein identified two candidates: AST96274.1 (682 aa; 53.9% identity) and AST98362.1 (689 aa; 39% identity) (Figure S2). Across five other *S. clausii* genomes (including the industrial strain KSM‐K16; RefSeq NC_006582.1), orthologs of both loci were detected (Figure S2). Phylogenetic analysis (Figure S3) showed that AST96274.1 clusters with *B. subtilis* GanA and the high‐identity *S. clausii* orthologs (96.6%–100% identity), while AST98362.1 is more distantly related and forms a separate clade, identifying AST96274.1 as the most likely GanA ortholog. Accordingly, a targeted deletion of the *S. clausii lacA* gene (locus tag bc8716‐10090) was performed. A linear DNA cassette containing *rfp* and *spec* ^*r*^ sequences was designed for site‐specific integration into the *lacA* locus of the *S. clausii* genome (Figure 1A) and subsequently employed to establish an antibiotic‐based transformation protocol.

To introduce the *lacA* deletion cassette into *S. clausii* DSM 8716, we tested the widely adopted natural competence protocol developed for *B. subtilis* (Yasbin et al. 1975); however, it did not yield any transformant in *S. clausii* DSM 8716 (data not shown). This result is consistent with the findings of Kovács et al. (2009) who reported that although genes associated with natural competence are present in *S. clausii* KSM‐K16, a strain closely related to DSM 8716, the absence of the regulatory gene *comK* likely renders the DNA uptake machinery inactive or severely impaired. Given these limitations, we chose to employ an electroporation protocol based on an optimised trehalose–mannitol hyperosmotic buffer (Zhang et al. 2015). Using this protocol, we routinely achieved transformation efficiencies of 7 ± 3 × 10^3^ CFU μg^−1^ of linear DNA, which is sufficient to support routine genome engineering in *S. clausii* DSM 8716.

The successful integration of the *rfp‐spec* ^*r*^ cassette at the *lacA* locus was confirmed by PCR amplification of the *rfp* and *spec* ^*r*^ sequences. Using primer pairs annealing to genomic regions flanking *lacA*, a longer amplicon was obtained in the mutant strain compared to the wild‐type strain. Furthermore, amplification products generated with primer pairs specific to each marker gene were exclusively detected in the engineered strains, confirming successful cassette insertion. In contrast, no amplification was observed in wild‐type controls, indicating the absence of the cassette (Figure 1B). Of the 15 colonies screened, 8 (54%) displayed the expected amplification pattern, indicating correct cassette integration. Sanger sequencing of the homology‐arm junctions confirmed precise integration and verified sequence fidelity at the insertion site.

Disruption of *lacA* was assessed using two phenotypic assays. Growth was evaluated on M9 minimal medium supplemented with either glucose or lactose as the sole carbon source (Figure 1C). Both WT and ∆*lacA::rfpspec* ^*r*^ strains grew on glucose, but only the wild type formed colonies on lactose, consistent with the inability of the Δ*lacA* mutant to catabolise this sugar. In parallel, wild‐type and recombinant colonies were streaked on LB agar containing X‐gal (Figure 1D). Wild‐type colonies appeared blue, indicating functional β‐galactosidase activity, whereas recombinant colonies were white, confirming loss of the *lacA* gene.

The replacement of the *lacA* coding sequence with the *rfp* gene enabled the use of the fluorescent protein as a transcriptional reporter for monitoring gene expression levels. As shown in Figure 1E, the recombinant strain exhibited a modest but statistically significant increase in fluorescence compared to the wild‐type strain grown in LB alone (*p* < 0.05). Notably, in the recombinant strain, RFP fluorescence approximately doubled in the presence of lactose.

### 3.2 Development of a Temperature‐Sensitive Vector for *S. clausii* Genetic Modification

**FIGURE 2:**
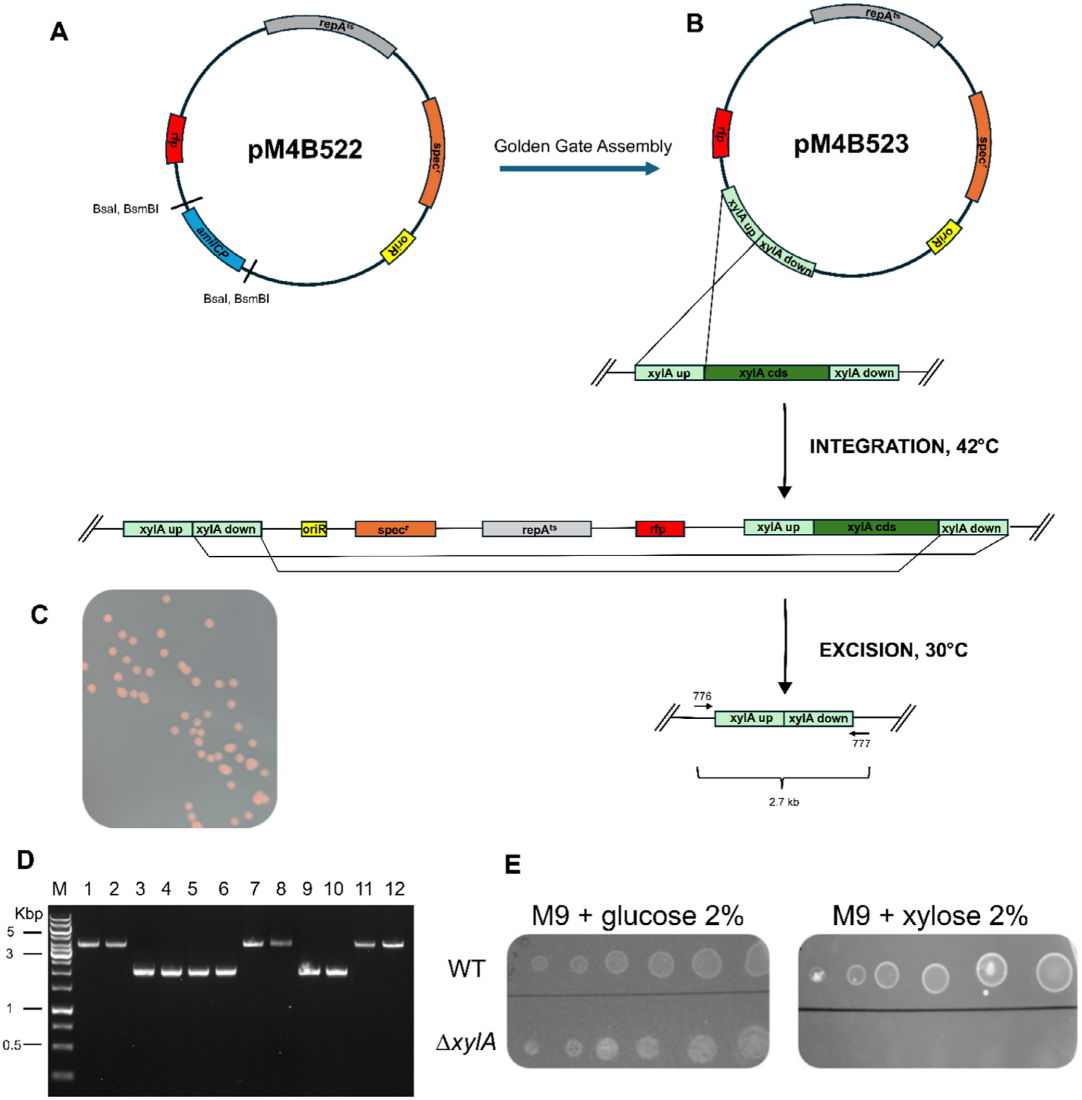
(A) Map of the pM4B522 plasmid. The plasmid carries an *E. coli* origin of replication (*oriR*, yellow), spectinomycin resistance cassette (*spec* ^*r*^, orange), the temperature‐sensitive replication initiator *repA* ^*ts*^ (grey) and the *rfp* reporter gene (red). The backbone also includes an AmilCP chromoprotein scaffold (blue), flanked by inward‐facing BsaI/BsmBI recognition sites to facilitate modular Golden Gate cloning, which allows cassette excision and scar‐free insertion of different DNA fragments; loss of AmilCP produces white colonies in *E. coli* . (B) Two‐step, markerless deletion of *xylA* in *S. clausii* DSM 8716 using the temperature‐sensitive shuttle vector pM4B523. Following Golden Gate Assembly, AmilCP was substituted with 1 kb homology arms (light grey) positioned upstream and downstream of the chromosomal xylA coding region (green). (i) Integration step: A single crossover between either arm and the chromosome inserts the entire plasmid, yielding a merodiploid intermediate. (ii) Resolution step: The second crossover can occur on either arm, yielding either the desired Δ*xylA* allele or restoration of the wild‐type allele. In both cases, curing resulted in spectinomycin‐sensitive clones, which were distinguished through colony PCR. (C) Plate showing *S. clausii* red colonies grown at 30°C on selective medium containing spectinomycin after transformation with the recombinant plasmid pM4B523. (D) Agarose gel electrophoresis of colony PCR products from *S. clausii* strains. Lane M: DNA ladder; Lane 1: WT control; Lanes 2–12: Recombinant ∆*xylA* clones. Lanes with the Δ*xylA*‐size amplicon (2.0 kb) correspond to deletion mutants; lanes with the WT‐size amplicon (4.0 kb) are revertants (wild type after resolution). (E) Carbon source utilisation assay for the Δ*xylA* mutant.

Having first established an efficient transformation protocol for *S. clausii* DSM 8716, we next developed a new temperature‐sensitive *E. coli–S. clausii* shuttle vector, called pM4B522. The new construct comprises (Figure 2A): (i) the temperature‐sensitive *repA* ^*ts*^ a replication origin from pE194—obtained from the pMAD vector—which supports replication in Gram‐positive hosts at 30°C but is lost at 42°C (Arnaud et al. 2004); (ii) the ColE1/pM4B1 origin for high‐copy replication in *E. coli* ; (iii) a spectinomycin‐resistance (*spec* ^*r*^) cassette from pM4B372 (developed in this study) for selection in both *E. coli* and *S. clausii* ; (iv) an *rfp* gene from pBs1C‐RFP (Radeck et al. 2013) included as a visual marker to assess transformation in Gram‐positive hosts; (v) dropout cassettes encoding the chromogenic protein AmilCP from plasmid JME5599 (Vidal et al. 2023) flanked by BsaI and BsmBI type IIS restriction sites, which are replaced during Golden Gate cloning and enable blue/white screening in *E. coli* .

The size of pM4B522 was reduced to 6888 bp, significantly smaller than the approximately 9 kb of other temperature‐sensitive vectors (Arnaud et al. 2004), to enhance ease of manipulation and improve transformation efficiency.

### 3.3 The pM4B522 Plasmid Enables Efficient Gene Deletion

To delete *xylA* in *S. clausii* DSM 8716, AmilCP in plasmid pM4B522 was replaced with two homology arms flanking the *xylA* coding region, generating plasmid pM4B523 (Figure 2B).

Following electroporation and 48 h incubation at 30°C, the resulting colonies (10^3^ colonies μg^−1^ plasmid DNA) were uniformly red and spectinomycin resistant due to plasmid‐borne RFP expression and the spectinomycin resistance cassette (Figure 2C).

PCR amplification using primers targeting the plasmid backbone yielded a product using DNA extracted from red transformants as template. In contrast, no amplification was observed in the white, spectinomycin‐sensitive colonies obtained at the end of the pop‐in/pop‐out protocol, indicating the absence of the plasmid‐borne spectinomycin resistance cassette (data not shown). Among the eleven ∆*xylA* candidate clones tested, six (55%) produced shorter PCR amplicons than the wild‐type when using primers positioned outside the homology arms. This result is consistent with the anticipated size reduction following the deletion of the *xylA* coding region (Figure 2D).

Sanger sequencing verified the precise, scarless deletion of the *xylA* coding region (data not shown).

On M9–xylose minimal medium (both solid and liquid), the Δ*xylA* strains exhibited no growth, while their growth on M9–glucose remained unaffected (Figure 2E). This indicates that the *xylA* gene is essential for xylose catabolism in *S. clausii* DSM8716, consistent with findings in *B. subtilis* (Gärtner et al. 1988).

**FIGURE 3:**
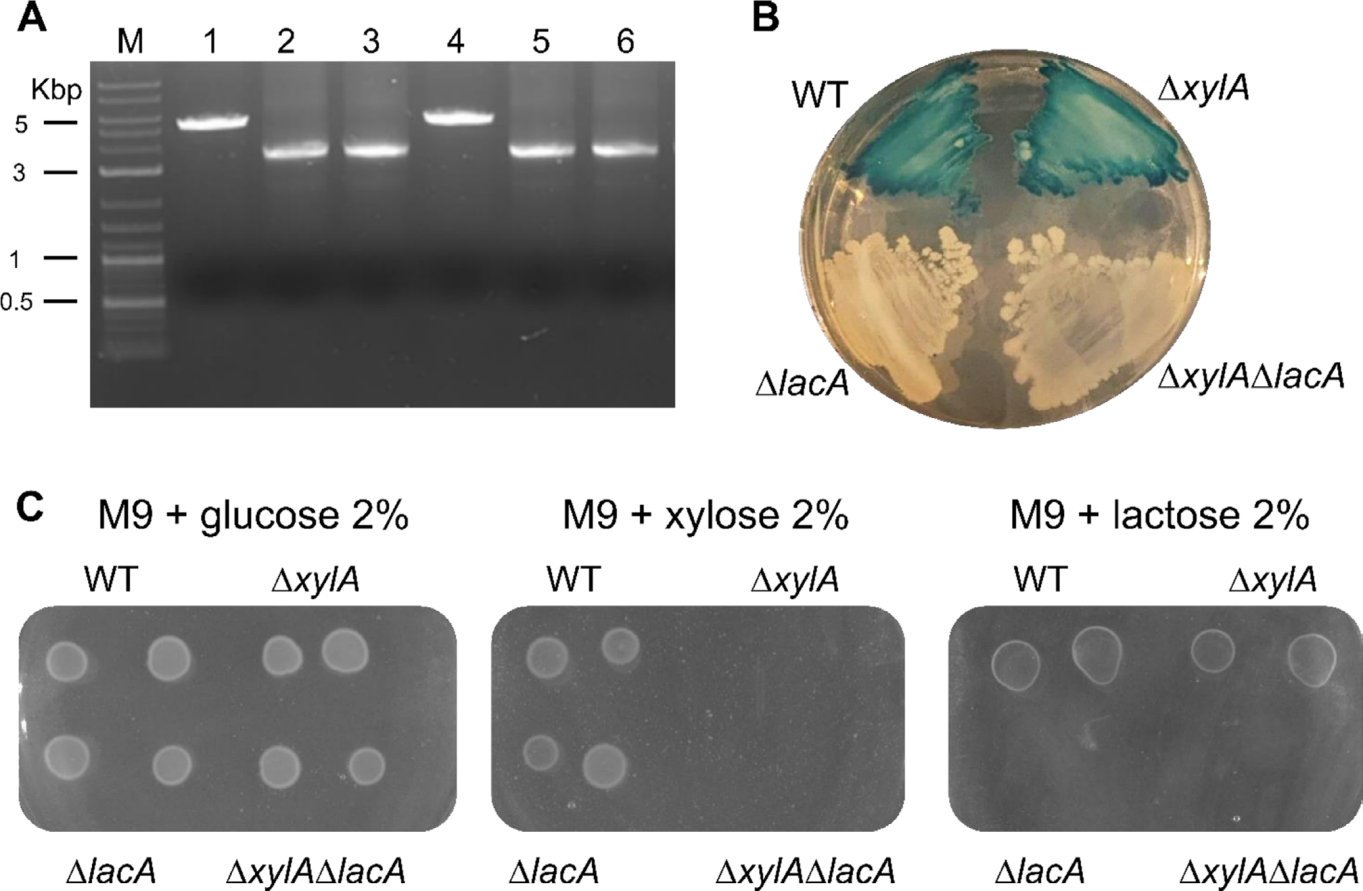
Molecular and phenotypic validation of *lacA* deletion. (A) Agarose gel electrophoresis of colony PCR products from WT and recombinant *S. clausii* strains. Amplification with primers flanking the *lacA* locus yielded a 5.2 kb fragment in the WT strain (Lane 1) and a 3.2 kb fragment in recombinant clones with the *lacA* deletion (Lanes 2–6; with one negative clone in lane 4). Lane M: DNA ladder (1 kb Plus Ladder, NEB). (B) Growth of *S. clausii* strains on X‐gal LB agar plate. (C) Growth of *S. clausii* strains on M9 minimal medium supplemented with either glucose (left), xylose (centre) or lactose (right) as the sole carbon source.

To demonstrate the reusability of pM4B522 for sequential genome editing, the *lacA* coding region was deleted in the Δ*xylA* background using the plasmid pM4B551, containing sequences flanking the *lacA* locus. Following transformation of *S. clausii* with pM4B551, white, spectinomycin‐sensitive colonies were screened using primers flanking the recombination site. The parental strain yielded a PCR product of the expected size, whereas four out of five Δ*lacA* candidates produced a smaller amplicon (Figure 3A), consistent with successful deletion of the *lacA* coding region. As previously observed, Sanger sequencing confirmed a precise, scar‐free deletion (data not shown).

Phenotypically, the Δ*xylA*Δl*acA* strain failed to grow when xylose or lactose was the sole carbon source and formed white colonies on LB–X‐gal plates, consistent with loss of β‐galactosidase activity from the *lacA* locus (Figure 3B,C).

For comparison, the same *lacA* deletion was performed in the wild‐type DSM 8716 background, yielding identical results on the level of genotype and phenotype.

### 3.4 pM4B522 Can Be Used for Insertion of Heterologous Genes

**FIGURE 4:**
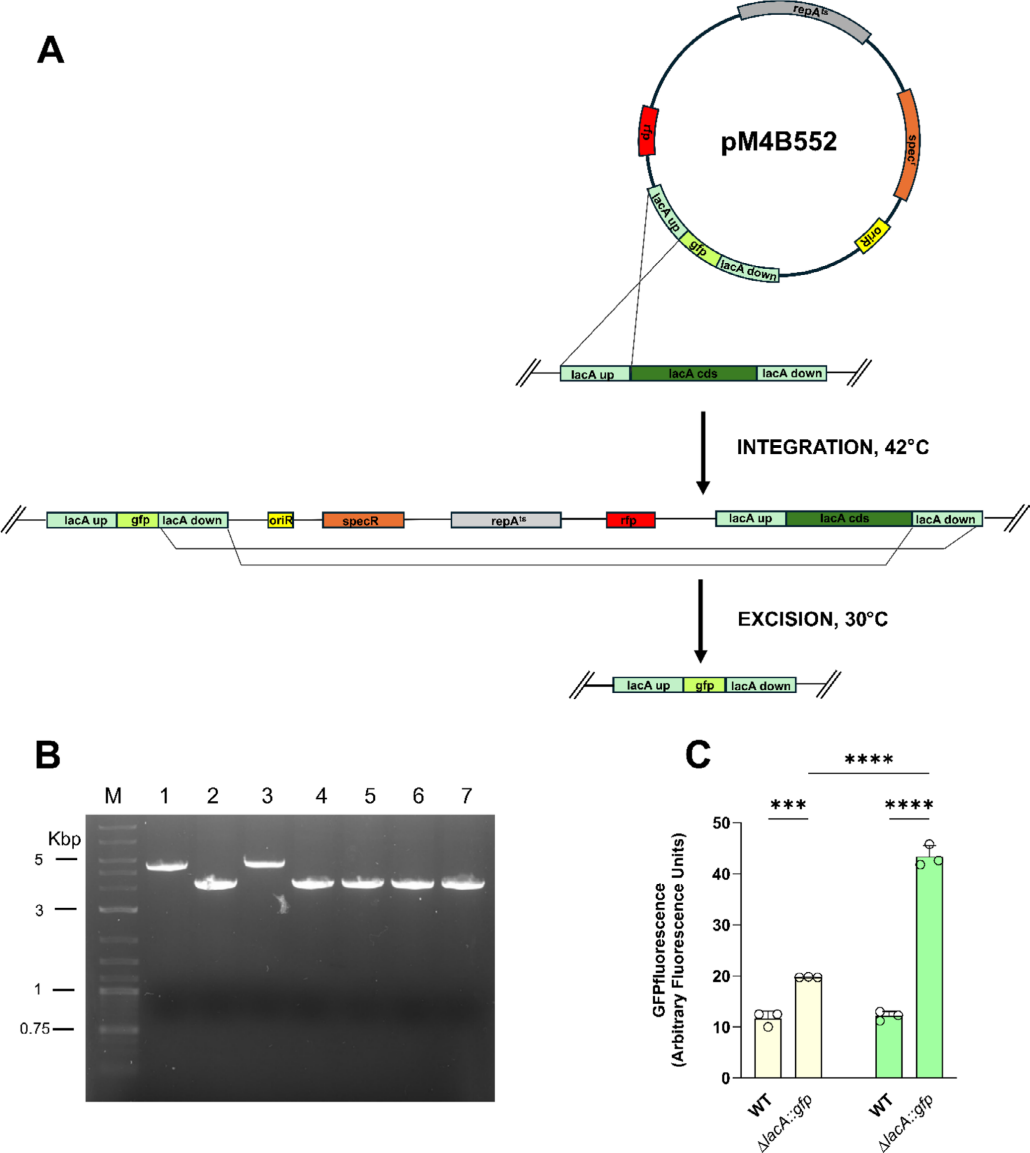
Replacement of *lacA* coding sequence with *gfp* in *S. clausii* DSM 8716. (A) Two‐step, markerless replacement of *lacA* with the GFP coding sequence using the temperature‐sensitive shuttle vector pM4B552. Upstream and downstream 1 kb homology arms (light green) flanking the chromosomal *lacA* coding sequence (green); in the pM4B552 plasmid, these homology arms flank the *GFP* coding sequence. (i) Integration step: A single crossover between either arm and the chromosome inserts the entire plasmid, yielding a merodiploid intermediate. (ii) Resolution step: A second recombination between the duplicated arms excises the vector backbone, leaving *gfp* at the locus. Black arrows indicate the direction of homologous recombination. (B) Agarose gel electrophoresis of colony PCR products from *S. clausii* strains. Amplification with primers flanking the *lacA* locus yielded a 4.8 kb product in the wild‐type strain (Lane 1) and a 3.5 kb fragment in recombinant clones containing the integrated *gfp* gene (Lanes 2–7), confirming successful replacement in lanes 2, 4, 5, 6 and 7 (80% positive). Lane M: DNA ladder. (C) GFP fluorescence in *S. clausii* strains after 24 h growth under different conditions. Fluorescence intensity of GFP expressed Δ*lacA::gfp* cells grown in LB (Light yellow bars) and in LB supplemented with lactose (Light green bars). Bars represent mean ± SD of three independent experiments. Statistical analysis was performed using one‐way ANOVA with Tukey's post‐test (***p < 0.001; ****p < 0.0001).

To evaluate the insertion of a heterologous gene, AmilCP coding sequence in pM4B522 was replaced by the *gfp* gene (Wicke et al. 2017). The *gfp* sequence was flanked by homology arms corresponding to the genomic regions upstream and downstream of the *lacA* coding sequence, resulting in the construction of plasmid pM4B552. The plasmid was introduced into *S. clausii* and, after the pop‐out step, white, spectinomycin‐sensitive colonies were screened by colony PCR. Of the seven recombinant clones tested, six displayed a PCR band of the expected size (Figure 4B). Sanger sequencing of three independent clones confirmed the precise replacement of the *lacA* coding sequence with *gfp*.

**FIGURE 5:**
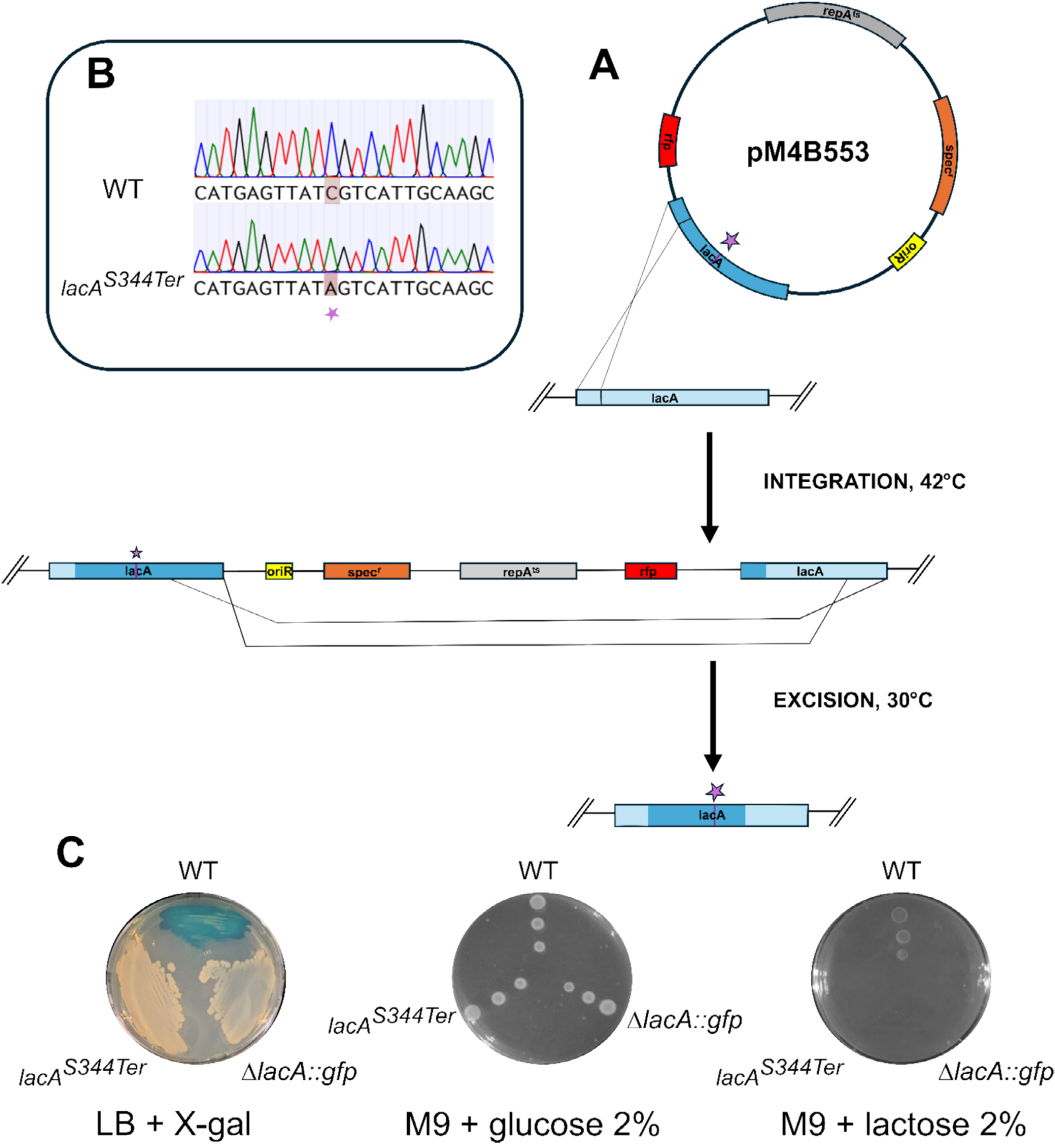
Allele replacement in the *lacA* coding sequence of *S. clausii* DSM 8716. (A) Two‐step, markerless allele replacement in the *lacA* coding sequence using the temperature‐sensitive shuttle vector pM4B553. (i) Integration step: A single crossover between either arm and the chromosome inserts the entire plasmid, yielding a merodiploid intermediate. (ii) Resolution step: A second recombination between the duplicated arms excises the vector backbone. The introduced point mutation is indicated by the purple star. Black arrows indicate the direction of homologous recombination events. (B) Sanger sequencing confirmation of the *lacA* C‐to‐A point mutation. Electropherograms of the strand for the parental strain (top) and the mutant clone (bottom). The highlighted region shows the single C‐to‐A transversion that converts the sense codon TCG (serine) into the in‐frame stop codon TAG (stop codon), introducing a premature termination within the *lacA* coding sequence. (C) Phenotypic controls of ∆*lacA::gfp* and *lacA* ^*S344Ter*^ recombinant clones. LB agar plate supplemented with X‐gal showing colony colour phenotypes. Wild‐type (WT) strain appears blue due to functional *lacA*, while *lacA* ^*S344Ter*^ mutant and Δ*lacA*::*gfp* mutants appear white, confirming replacement or inactivation of the *lacA* gene. Growth on M9 minimal medium supplemented with glucose (left) or lactose (right).

These findings were further supported by phenotypic assays (Figure 5C). Fluorometric analysis showed that GFP fluorescence was approximately twofold higher in the recombinant strain compared to the wild type. Supplementation of LB medium with lactose led to an approximately 1.8‐fold increase in reporter fluorescence in the recombinant strain compared to LB alone, and nearly a threefold increase relative to the wild‐type strain (Figure 4C).

### 3.5 The pM4B522 Plasmid Enables Allele Replacement

To evaluate the tool's ability to introduce single‐nucleotide changes into the bacterial genome, a mutated version of the *lacA* coding sequence carrying a C‐to‐A transversion, converting codon 344 (TCG, serine) into the stop codon TAG, was inserted into plasmid pM4B522, resulting in the construction of plasmid pM4B553 (Figure 5A). Following the integration steps, Sanger sequencing of white, spectinomycin‐sensitive transformants revealed a single C‐to‐A substitution in eight out of twelve clones analysed, resulting in the introduction of an in‐frame stop codon (Figure 5B). Phenotypic assays showed that the *lacA* ^S344Ter^ mutants remained white on LB–X‐gal plates and failed to grow when lactose was the sole carbon source, whereas growth on glucose was indistinguishable from the parental strain (Figure 5C).

### 3.6 Portability of the pM4B522 Platform to *Bacillus subtilis* 168

**FIGURE 6:**
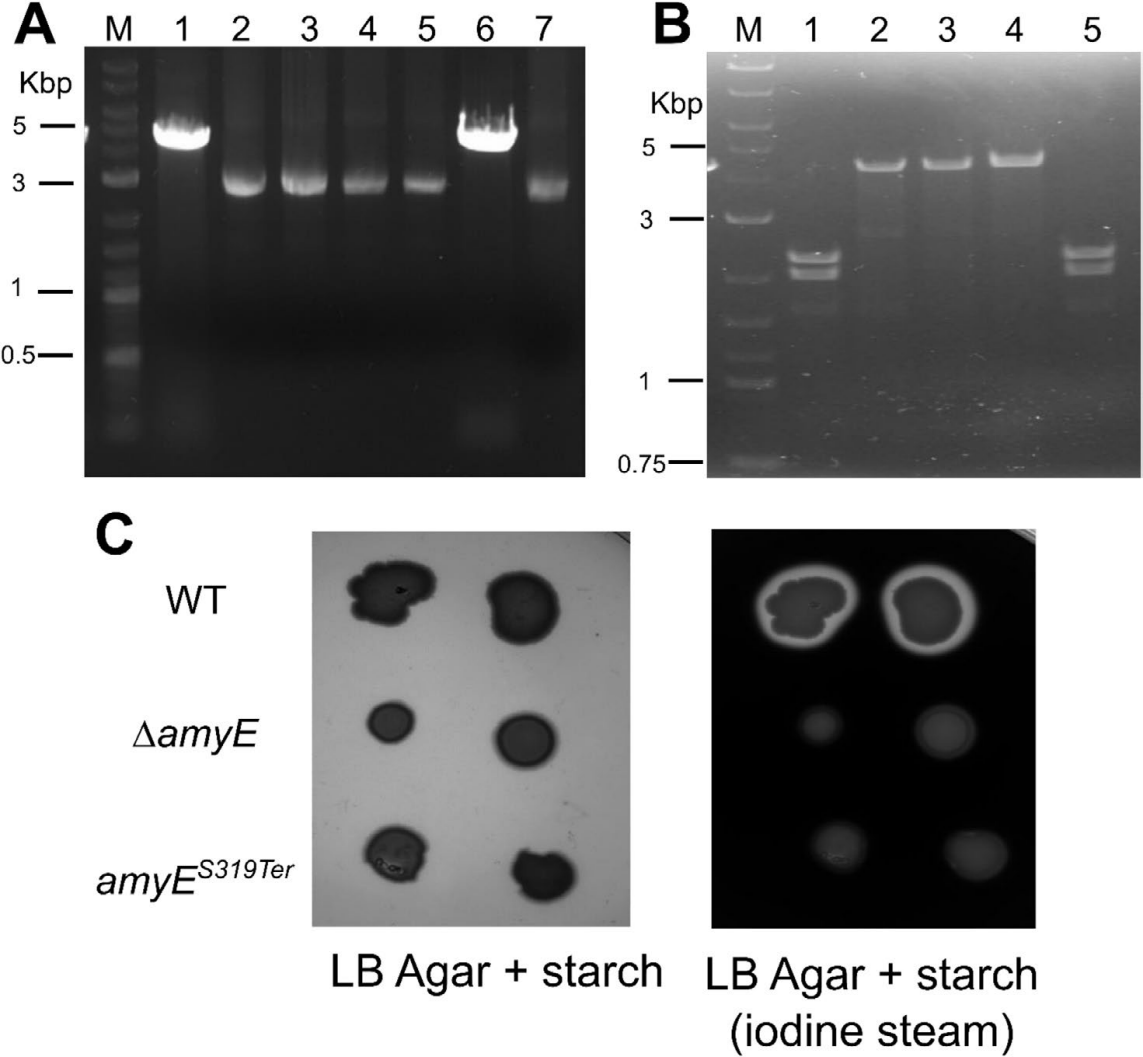
Markerless gene deletion and allelic replacement in *B. subtilis* 168. (A) Agarose gel electrophoresis of colony PCR products from *B. subtilis* strains *ΔamyE*. Amplification with primers flanking the *amyE* locus yielded a 4.7 kb product in the wild‐type strain (Lane 1) and a 2.7 kb fragment in recombinant clones (Lanes 2–7), confirming successful replacement in lanes 2,3, 4, 5 and 7. Lane M: DNA ladder. (B) Agarose gel electrophoresis of colony PCR products from *B. subtilis* strains digested with SalI. Lane M: DNA ladder; Lane 1: WT control; Lanes 2–5: recombinant *amyE* ^*S319Ter*^ clones. Positive clones (lanes 2, 3 and 4) were not digested by SalI, while the WT and a negative clone (lane 5) showed two bands after digestion. (C) Starch hydrolysis assay (*amyE* test) on LB agar plates supplemented with starch. WT and mutant strains were streaked onto starch‐containing LB plates. After overnight incubation, the plates were photographed (left panel) prior to exposure to iodine vapour (right panel), which interacts with starch. The presence of a clear halo surrounding colonies indicated starch degradation. Only the WT strain produced such a halo, confirming functional amylase activity. In contrast, neither the *ΔamyE* knockout strain nor the *amyE* ^*S319Ter*^ mutant showed halo formation, indicating loss of starch‐degrading capability due to deletion or inactivation of the *amyE* gene.

To evaluate whether the marker‐free system developed for *S.clausii* could be transferred to other Gram‐positive hosts, we tested it in *B. subtilis* 168, targeting the chromosomal *amyE* locus. The plasmid pM4B554, harbouring homology arms flanking the *amyE* coding sequence, was introduced into *B. subtilis* . Following the pop‐in/pop‐out recombination steps, white, spectinomycin‐sensitive colonies were screened by PCR. In five out of six clones (80%), a smaller amplicon was obtained compared to that of wild‐type cells, indicating successful deletion of the target region (Figure 6A). Sanger sequencing across both recombination junctions confirmed the scar‐free deletion of *amyE* (data not shown). Loss of α‐amylase activity was verified by a starch hydrolysis assay (Figure 6C).

To enable single‐nucleotide editing in *B. subtilis* , plasmid pM4B555 was engineered to include the full *amyE* coding sequence containing a targeted C‐to‐A transversion at nucleotide position +956 (codon 319), which disrupts a SalI restriction site (changing GTCGAC to GTAGAC) and introduces an in‐frame stop codon (TAG), replacing the original serine residue.

After the recombination pop‐out step, colony PCR using primers flanking the recombination sites produced a fragment consistent with the wild‐type length. Of the four amplified fragments tested, three (75%) resisted SalI digestion indicating the absence of the SalI site and confirming successful genetic modification (Figure 6B). Sequencing of these PCR products (data not shown) confirmed the presence of the intended single‐base edit. These results were further validated by a phenotypic assay of amylase activity (Figure 6C).

## 4 Discussion

Two major obstacles have long hindered the use of *S. clausii* not only as a spore‐forming chassis for biotherapeutic delivery, but also in broader industrial and environmental biotechnology applications: (i) a highly cross‐linked peptidoglycan layer that restricts DNA uptake (Donovan et al. 1987) and the absence of the competence master regulator ComK, which likely precludes efficient natural transformation (Alcaraz et al. 2010); (ii) the lack of a molecular tool for manipulation of its genome.

By combining a trehalose–mannitol hyperosmotic buffer with mild cell‐wall‐weakening agents, we routinely achieved transformation efficiencies of approximately 7 × 10^3^ CFU/μg DNA, an order of magnitude higher than those previously reported for recalcitrant bacilli such as *Bacillus pumilus* (Danilova et al. 2022), and comparable to or exceeding those seen in *B. thuringiensis* (Lereclus et al. 1989). While the transformation efficiency remains below the ≥ 10^7^ CFU μg^−1^ reported for response‐surface‐optimised protocols in *B. subtilis* ZK (Zhang et al. 2015), this study presents, to the best of our knowledge, the first successfully established protocol for genetic transformation in *S. clausii* DSM 8716. Further studies are required to investigate natural competence in *S. clausii* . To this end, introducing regulatory elements such as the *comK* gene and the competence‐stimulating peptide ComS from *B. subtilis* , which stabilises the master regulator ComK, could help to evaluate their impact on natural competence in *S. clausii* .

To address the lack of molecular tool for genome manipulation in *S*. *clausii*, we developed a recyclable, marker‐free genome‐editing system built on a new temperature‐sensitive vector (pM4B522). This plasmid retains the pE194‐derived *repA* ^*ts*^ module from pMAD (Arnaud et al. 2004) while all other functional elements were redesigned specifically for efficient genetic engineering in *S. clausii* . The pM4B522 vector has a size of 6.9 kb, making it significantly smaller than pMAD and other temperature‐sensitive vectors such as pKSV7 (Gao et al. 2017) and pKOR1 (Bae and Schneewind 2006). Additionally, the approx. 1 kb AmilCP cassette is removed during Golden Gate assembly, reducing the final construct size to 5.8 kb plus the homology arms. The reduced plasmid size improves electroporation efficiency in *Bacillus* species (Ohse et al. 1995).

The AmilCP chromogenic drop‐out reporter is flanked by BsaI/BsmBI Type IIS restriction sites, strategically positioned so that enzymatic digestion removes both the reporter and the recognition sequences. This configuration generates predefined overhangs on the vector backbone, enabling seamless insertion of DNA fragments through single‐step Golden Gate assembly.

Screening is visual in both hosts: in *E. coli* , loss of *amilCP* produces white colonies, whereas in Gram‐positive transformants the *rfp* reporter turns single‐crossover colonies red making the curing step unambiguous. Once the plasmid has been excised following a second crossover event, no antibiotic marker remains in the chromosome; consequently, the same *spec* ^*r*^ backbone can be recycled indefinitely without changing antibiotics and iteratively across multiple rounds of genetic modification. Since pM4B522 retains the pE194‐derived *repA* ^*ts*^ origin of replication, it is expected to be compatible with hosts that support this replicon, making it potentially portable across a wide range of low‐GC Gram‐positive bacteria where pMAD is known to replicate (Monk and Foster 2012; Abi Khattar et al. 2009).

Several genome editing strategies, including scar‐free approaches, have been developed for *Bacillus* spp. However, existing tools present limitations not observed with our system. For example, (i) despite > 95% efficiency (Altenbuchner 2016; Westbrook et al. 2016; Zou et al. 2022), CRISPR/Cas9 may introduce off‐target mutations, and its expression can be cytotoxic (Kim et al. 2025), (ii) Site‐specific recombinases (Cre–loxP, Xer/dif, FLP‐FRT) leave recombination scars (Yan et al. 2008), (iii) counter‐selectable metabolic markers (e.g., upp with 5‐fluorouracil) require host‐specific optimisation and often result in lower recombination efficiencies (Fabret et al. 2002) and (iv) RecT‐mediated recombineering in *Bacillus* is largely restricted to specialised strains expressing heterologous annealases (Sun et al. 2015).

Using the pM4B522 system, genome editing was achieved through homologous recombination and a temperature‐sensitive replicon, without leaving behind foreign genes, residual recombinase sites, or acquired antibiotic resistance markers. Once the plasmid pop‐out step has occurred, no residual vector‐derived DNA sequences remain in the host genome, apart from the intended genetic modification. This marker‐free, scarless system aligns with the US FDA's Chemistry, Manufacturing and Control guidance for live biotherapeutics, which strongly discourages the presence of acquired resistance genes (FDA—*Early Clinical Trials with Live Biotherapeutic Products: Chemistry, Manufacturing, and Control Information; Guidance for Industry*).

The newly developed system has been successfully employed to carry out three types of genome editing strategies in *S. clausii* DSM 8716: (i) markerless deletions of *xylA* (xylose isomerase) and *lacA* (β‐galactosidase); (ii) introduction of a premature stop codon in *lacA*; and (iii) insertion of coding sequences for heterologous proteins such as RFP and GFP under lactose‐inducible regulatory elements. However, the observed expression levels were relatively low, highlighting the need to identify and characterise promoters capable of driving robust heterologous gene expression in *S. clausii* . Similar efforts in *Bacillus* spp. have led to the development of synthetic promoter libraries, which could potentially be adapted for use in *S. clausii* (Okay 2024; Hammer et al. 2006).

Using this tool, we demonstrated through targeted gene deletions and stop codon insertions that LacA and XylA are essential for lactose and xylose metabolism in *S. clausii* DSM 8716, underscoring the system's utility for functional genomics research.

From an application perspective, *S. clausii* spores withstand gastric acidity, germinate in the small intestine, and have an excellent human safety record (Acosta‐Rodríguez‐Bueno et al. 2022). Engineering this chassis opens the door to in situ delivery of bioactive molecules, from anti‐inflammatory cytokines to metabolic hormones–an approach already validated in *Lactococcus lactis* (Steidler et al. 2003) and *E. coli* Nissle (Isabella et al. 2018). Since pM4B522 introduces only the intended genetic modification without leaving behind any foreign DNA, it could significantly reduce both regulatory and technical hurdles for implementing protein‐secretion strategies in an *S. clausii* background. By combining the strain's inherent antibiotic resistance with newly developed genetic engineering capabilities, this approach positions it as a strong candidate for next‐generation live biotherapeutic development.

In conclusion, the electroporation protocol developed in combination with the recyclable Golden‐Gate vector pM4B522 provides a robust and flexible platform for genetic manipulation of *S. clausii* DSM 8716, converting it from a genetically recalcitrant strain into a tractable chassis for synthetic biology. The successful implementation of this tool in *B. subtilis* 168 highlights its potential for broader application across other Gram‐positive bacteria.

## Author Contributions

**Claudia Cappella:** conceptualisation, investigation, writing – original draft, writing – review and editing, visualisation. **Carsten Jers:** conceptualisation, writing – Review and Editing. **Lorenzo Ninivaggi:** investigation. **Maurizio Bettiga:** writing – review and editing. **Ivan Mijakovic:** funding acquisition, writing – review and editing. **Gennaro Agrimi:** funding acquisition, conceptualisation, visualisation, writing – original draft, writing – review and editing, supervision, data curation. **Pasquale Scarcia:** conceptualisation, writing – original draft, writing – review and editing, supervision, data curation.

## Funding

This work was supported in part by grants from the Novo Nordisk Foundation (NNF20CC0035580) and the SMARTNUTRIHEALTH 2—Progetto N.Pos. 95—N.ro MISE F/310095/01/X56—Accordi di Innovazione—D.M. 31 Dicembre 2021 e D.D. 18 Marzo 2022—CUP: B49J25000040005. Funding was also provided by the European Union's Horizon Europe program under the call HORIZON‐WIDERA‐2023‐ACCESS‐02, Grant Agreement No. 101159570 (Twinn4MicroUp).

## Conflicts of Interest

The authors declare no conflicts of interest.

## Supporting information


**Data S1:** mbt270287‐sup‐0001‐DataS1.docx.

**Data S2:** mbt270287‐sup‐0002‐DataS2.gbk.

## Data Availability

The data that support the findings of this study are openly available in Zenodo at https://zenodo.org/records/17404389, reference number https://doi.org/10.5281/zenodo.17404388.
