# Supplementary material for: Development of a Tool for High‐Efficiency, Markerless and Iterative Genome Editing in Shouchella clausii
Source: Microb Biotechnol. 2026 Feb 9;19(2):e70287. doi: 10.1111/1751-7915.70287 (PMC12885165; doi:10.1111/1751-7915.70287)

**Supplementary materials**

**Table S1. List of primers used in this work**

| **Name** | **Sequence** | **Use** |
| --- | --- | --- |
| 757 | aaagaattcttgacatttttcttgtggatctgtataataaagaataatta | Amplification of *spec^r^* cassette – forward primer with EcoRI site |
| 758 | atactcgagatcgatcaatagttacaaattgtttcacta | Amplification of *spec^r^* cassette – reverse primer with + XhoI site |
| 807 | agtttgcgtggcaaatgg | *lacA* upstream region – forward primer |
| 50 | acgtcttcggaggaagccacgccaagcgattgtcgt | *lacA* upstream region – reverse primer, overlaps with *rfp* |
| 51 | acgacaatcgcttggcgtggcttcctccgaagacgt | *rfp* – forward primers, overlaps with *lacA* up |
| 52 | ctctcctttctcgcctgcttaagcaccggtggagtgacg | *rfp* – reverse primer, overlaps with *specR* |
| 716 | gcaggcgagaaaggagaggagggaggaaaggcagga | *spec^r^* – forward primer (1) |
| 717 | cgaggctcctgtcactgccgccgtatctgtgctctc | *spec^r^* – reverse primer (1) |
| 809 | gcagtgacaggagcctcgatgtcattaatgactgctg | *lacA* downstream region – forward primer, overlaps with *specR* |
| 810 | tcaatcttaccaaaacccacc | *lacA* downstream region – reverse primer |
| 805 | tggaggtgcggatgtgaaag | Nested forward primer for overlap extension PCR |
| 806 | ctcagccacttcaaagtcc | Nested reverse primer for overlap extension PCR |
| 803 | gatttcgattcctgttgccc | *lacA* deletion check (control) - forward primer for colony PCR |
| 804 | ccgcttcaataagatgctctac | *lacA* deletion check (control) - reverse primer for colony PCR |
| 1015 | ggctacggtctcaggagccaaaccagccagctccgtatgc | *xylA* upstream region – forward primer with BsaI site |
| 18 | ctttgctgtttttcttctgctcaaagtagacgatcggc | *xylA* upstream region – reverse primer, overlaps with *xylA* down |
| 1016 | ggctacggtctccatggcaacagtaagtgaaacgtcagtcaccc | *xylA* downstream region – reverse primer with BsaI site |
| 19 | gccgatcgtctactttgagcagaagaaaaacagcaaag | *xylA* downstream region – forward primer, overlaps with *xylA* up |
| 775 | agtaagtgaaacgtcagtcacc | *xylA* deletion check (control) - reverse primer for colony PCR |
| 777 | cgccggatagacttgtttatac | *xylA* deletion check (control) – forward primer for colony PCR |
| 747 | gcaatgccaaccatacgcctgcaacc | *xylA* sequencing primer |
| 53 | ctgcaggaaggtttaaacgcatttagg | Plasmid sequencing primer |
| 54 | gaaattaatacgactcactataggg | Plasmid sequencing primer |
| 1040 | gctacggtctcaggaggaggtgcggatgtgaaagcagc | *lacA* upstream – forward primer with BsaI site |
| 1041 | accaggtctcaccgttctcctactccttctcttcttgtcatttcagttgg | *lacA* upstream – reverse primer with BsaI site |
| 1042 | tggcggtctctacggttgtaaaagagatgaggctcattcgagg | *lacA* downstream – forward primer with BsaI site |
| 1043 | ggctacggtctccatggcctcagccacttcaaagtccaagc | *lacA* downstream -reverse primer with BsaI site |
| 1017gga | gctacggtctcaaatgcgtaaaggagaagaacttttcactgg | *gfp* – forward primer with BsaI site |
| 1018gga | gctacggtctcctagattatttgtatagttcatccatgccatgtgtaatc | gfp – reverse primer with BsaI site |
| 1050 | acttggtctcacattctcctactccttctcttcttgtcatttcagttgg | *lacA* upstream region – reverse primer with BsaI site |
| 1051 | ggtctcatctattgtaaaagagatgaggctcattcgagg | *lacA* downstream region – forward primer with BsaI site |
| 807 | agtttgcgtggcaaatgg | *gfp* insertion – colony pcr FOR |
| 810 | tcaatcttaccaaaacccacc | *gfp* insertion – colony pcr REV |
| 815 | ggaatgcgctatatccaaacagaagg | Sequencing across *lacA* locus |
| 816 | taggagttgcttgacttcttctttaagc | Sequencing across *lacA* locus |
| 1072 | tagaggtctctagtcattgcaagccgttgcccacg | *lacA* downstream region – forward primer introducing stop codon (A replaces C) with BsaI site |
| 1073 | aggtctctgactataactcatgcatgcctggcttcttgg | *lacA* upstream region – reverse primer introducing stop codon (T replaces G) with BsaI site |
| 1054 | ggaagtgctccgtgtcgaa | qPCR – forward primer for WT *lacA* |
| 1088 | gctacggtctcaggagttgatgggaatcacgagacag | *amyE* upstream region – forward primer with BsaI site |
| 1089 | accaggtctcaccgtcattcttgacactccttatt | *amyE* upstream region – reverse primer with BsaI site |
| 1090 | tggcggtctctacggtgagggcaaggctagacggg | *amyE* downstream region – forward primer with BsaI site |
| 1091 | ggctacggtctccatggatcggagtgagcgccacaagtg | *amyE* downstream region – reverse primer with BsaI site |
| 1097 | accaggtctctagacatggatgagcgatgatgatatc | *amyE* downstream region – forward primer introducing stop codon (A replaces C) with BsaI site |
| 1098 | tggtggtctctgtctactcttcatcatcattggcatac | *amyE* upstream region – reverse primer introducing stop codon (T replaces G) with BsaI site |
| 732 | cagtgccgtttaccgttcgcca | *amyE* deletion check (control) - forward primer for colony |
| 733 | atcggagtgagcgccacaagtg | *amyE* deletion check (control) - reverse primer for colony PCR |
| 718 | ttgatgggaatcacgagacag | *amyE* sequencing primer |
| 721 | aagttcagctcagtgatacctgc | *amyE* sequencing primer |
| 1121 | ataccaccagtgattatgccg | *amyE* sequencing primer |
| 1122 | aaacgccgtctctggtccattat | *amyE* sequencing primer |
|  | Koo BM, Kritikos G, Farelli JD, Todor H, Tong K, Kimsey H, et al. Construction and Analysis of Two Genome-Scale Deletion Libraries for Bacillus subtilis. Cell Syst. 2017 Mar;4(3):291-305.e7. | |

**S1. Construction of editing plasmids, diagnostic colony PCR, and junction sequencing**

**Δ*xylA* (plasmid pM4B523).** For deletion of *xylA*, the left arm was amplified with primers 1015/18 and the right arm with 1016/19, both from *S. clausii* genomic DNA. The two arms were inserted into pM4B522 by one-pot BsaI Golden Gate to yield pM4B523. *S. clausii* colonies were screened by colony PCR with external primers 775/777. Upstream and downstream junctions were verified by Sanger sequencing using primers 777 and 747, respectively.

**Δ*lacA* (plasmid pM4B551)**. For deletion of *lacA*, homology arms were generated with primer pairs 1040/1041 (left) and 1042/1043 (right) and assembled into pM4B522 by BsaI Golden Gate to obtain pM4B551. Recombinant *S. clausii* colonies were identified by colony PCR with external primers 803/804. Correct integration was confirmed by Sanger sequencing using primers 815, 816, 803, and 804.

***lacA* replacement with *gfp* (plasmid pM4B552).** For replacement of *lacA* with *gfp*, the left and right arms were amplified with 1040/1050 and 1051/1043, respectively; the *gfp* cassette was amplified from a Twist-synthesized fragment with primers 1017gga/1018gga. The three fragments (left arm–*gfp*–right arm) were assembled into pM4B522 by a single-pot BsaI Golden Gate reaction to generate pM4B552. *S. clausii* colonies were screened by colony PCR with external primers 807/810 and verified by Sanger sequencing with primers 815, 816, 803, and 804.

***lacA* allelic replacement (plasmid pM4B553)**. The left and right *lacA* homology arms were PCR-amplified from *S. clausii* DSM 8716 genomic DNA with primers 1040/1072 and 1073/1043, respectively. Fragments were assembled into pM4B522 by one-pot BsaI Golden Gate cloning. The single-nucleotide change (Ser-to-STOP; TCG-to-TAG at codon 344) was encoded directly in the complementary 4-nt BsaI overhangs at the junction between the two arms, so that the resulting plasmid specified the desired single-nucleotide edit without introducing scars. Junctions and the edited codon were verified by Sanger sequencing with primers 804, 815, 816, 1054, and 803.

**Δ*amyE* in *Bacillus subtilis* 168 (plasmid pM4B554).** To target the neutral *amyE* locus in *B. subtilis* 168, the left and right homology arms were PCR-amplified from *B. subtilis* genomic DNA with primers 1088/1089 (left) and 1090/1091 (right) and cloned into pM4B522 by BsaI Golden Gate assembly. Candidate *B. subtilis* colonies were screened by diagnostic PCR with the external primer pair 732/733 and confirmed by Sanger sequencing of both junctions using primers 718 and 721.

**Allelic replacement in *amyE* cds in *B. subtilis* 168 (plasmid pM4B555).** For a markerless single-nucleotide edit within *amyE*, the left and right homology arms were amplified with primer sets 1088/1097 and 1099/1091, respectively, and assembled into pM4B522 by BsaI Golden Gate. The programmed C-to-A transversion introduced a premature stop codon (TAG) at codon 319 of *amyE*; as above, the single-nucleotide variation was encoded in the complementary BsaI 4-nt overhangs at the arm junction, leaving no residual. Recombinants were amplified with the external primers 732/733 and the resulting amplicon was subjected to diagnostic SalI digestion to distinguish edited from wild-type alleles. Correctly edited clones were verified by Sanger sequencing with primers 1121 and 1122.

**Table S2. Sequences of homology arms**

| *xylA* upstream | ccaaaccagccagctccgtatgcagccagcatcgccgcacccatgcctggcccttgctcgcttgtctgcttcacaattggcgtttggaaaatgtcagcttgcatttgtagccacgtttcgctttttactcctccgccaatggaaacaattctctcaatcgtctttccttgttctctgaacaaatcaagtgactctcttaatgaaaaagtgatgccttcaaggatcgacctagtaaagtcggccttcgtatggctgctatcagcaccgataaaactagcacggatagtagcgtcagcgtgaggcgtccgctctccggcaagatagggcgtaaacaacaacccatttgcccctggggggacttctgccactcctgcaagcaattgcggaaacgtttcttcaggagcaaatgtttgcttaaaccaacttaaactatatcctgccgctaatgtgacgcccattgtataaaacgcatcttcagctgcatggttaaaataatggacgcctcctccaaatgtcttccctttttcttcctggtacgaaagcataacacctgacgtgccaatgctagcgagcgtcttgccgttttctaaaattccagctcctactgctccgcacgcattatcggctcctccagcaaaaacgggtgttttgcttggcagccccgtcgctaatgcgatttcttccgtcaactggccgacacaagcgtgtgattccactagcgtcggacaaagcgatggatcaatgccaaatgccgaacatagcggttcgctccatgtttttctttcgatatcaagcaagagtgtacctgctgcgtcagaatagtcggtatgcaatgcaccagtcaacttcaagcgcaaatagtcttttggaagcacgaatttggccgcttttgtaaacacttccggttcatgctcccgtacccacatcagctttggcaacgtaaaaccttcaagcgctggatttttggctaattgttgcagcctttcagcgcctagcttttcgtaaatcgctttgcattgcggggaggtgcgtgtatcattccataaaatcgccggacgcaacacttcgttttgttcatcgagcaagacgagcccatgcatttgccccgaaaaactgattccctcaatttgagtcgttgcttgtggcaaggctgctgctatgtccgataatccagcaacggtttccctgacccaatcgtttggatcttgctcgctccatcctgatttagggtgagacaacgggtatggtttagacacttccatgaccacttcacccgtctcgttaacgacaagcaatttcactgcgcttgtgcctaaatcaacaccaatgacatgtttcaatttgaatcgctccttgccgatcgtctacttt |
| --- | --- |
| *xylA* downstream | gagcagaagaaaaacagcaaagaaaatgggaatgcataggacgtctgcattccctcgtaaactatgtttttagctcgttcctgcctcgctctcctgtgcgttgatcctttcatttcagcacaatttggtaccgttccattttttcgctaatgtctgtaatcgcttcccgtaatagcggcaagcaggagtcaaaacgctcctcattaaagcgataggctgggccggatatagtaatggaaccaacgatccgcccttcatggaaaagcggcatcgccaaggcaatgacatcttccgtatattcgccatggctaatagcaaacccgcgtttgcgaatggttgccagctcattacgcaattcaatttcacttgtcatcgtcctgtctgtataggctttcaaacctcccgcaatcacttcctcaattgcctcgtcaggcatatacgccaagatgctgcggtaagacgcgcctacatataagggagcacggctgccagcagaaacggaaaacttcactttattggctggctcaaccacttcaagcgtaagcgcttctttgccgtccagaattgttagaaacgtggactcgcctgttttttccgttaaacgggcaaggcttgggcggatcaattcttttacatttaactggtcgtacataatcgaacctagctcccacacggcaaacccgaggctatatttctttgtctctgtatctttcgttagaaagttttgggcttccagtgtttcaagtatccgataaattttcgtatgattgatccccgtagcgttggacagctccctgcctccccaaacaggcttttctttcgtaaacatttttaaaatctccaatgcttgatgtacagtttttaacacagctcctcctaggccattgcggcatacttaatcattactaagtttacatcaaaacactgcgcatgggtgactgacgtttcacttact |
| *lacA* upstream | tggaggtgcggtgtgaaagcagcggttacagttgaaccaattgctggattggatgatcgctttattaaaggagccgatatctcgatgttgccagaaatcgaggaaaacggcgggcgttattatgccgatggagaacaaattgatccactcgtattgttgcaggaaaagggcgtaaatgctgtccgtatccgcatttgggtagatccttacgatgaaaacggcaacccgtatggcggagggactgttgatggacagcgcgccgttgatctggcaaagcgcgcccatagctatggttttcaactgttgctcgattttcactacagtgatttttggacagacccagggaagcagtttaagccaaaaagctgggaacattacagttttccggaattaaaggacgctatctatcaccatacatcaaatgtgcttggccagttaaaggatgcagggatcattccggaaatggtccaagttggcaatgaaattaatgccggaatgctatggcctgacggcaaaagctggggagaaggcggcggtgaatttgataggctcgccgcattgttgaaagcagggcttactgccgtgaaagactgcgacccgggtatccgtacgatgcttcatttagcagaaggcggagatatcgatatggctcaatggtggctcgatgagatcgggaagcgggacgttgattttgatgtcgttgggttgtcttactatccttactgggatggggatttcagtaagctcaaacaagtaatggagcttgttactaacgattatggcaaaggcgtcaatgtcgtcgaaacagcatatggctttacgacagacaatggcgacaacttagataatatcttttcatgtgaatacgcacagctagtcggttatccagcatcaccgcaagggcaagcgtcttacttgcgcgatttaatggcgacgattcacacggcaggaggagaagggttctattattgggaaccactttggattccagtgtctggcgcctcttgggcgaacgaagccggaatgcgctatatccaaacagaaggggaagttggcaatgcttgggacaatcaagcgatgtttaattttaatggagaagcattgccttcattagatgtgttccatttagtaaaccaactgaaatgacaagaagagaaggagtaggaga |
| *lacA* downstream | ttgtaaaagagatgaggctcattcgaggattttgagcataataaatatgctgcactataacgaatataatgagctgcttcgagtattactagtaaaacaacacataagaccgatagccctacacggctgccggtcttccttttttatacaggtagaaataagagggaaagaaattatttgatgttgggaaatatccaattaacttacattatcggaaaacatcgtctatactagtagtattatatattgatagaggtgatacgatgtcattaatgactgctgaaaaagctggggcgaaaattgttgagtggtatagttgtgtaattgctaagtctcatgatcaagctattttgcttaaagaagaagtcaagcaactcctaagtgagatgaaagacaatgataagatattggcctactattctttagttgaatttaggcatgacatgctgataaatagatacaacaaaaatgaattacaggctgattttcagaatattgaacacatagctaaaattgacaacatgttaaagtacctctattactttgttagtggtcaaagcgagtatgtcaatgaacgctatagatcagctattaagctttttaataaagctcagcgcttgttagagtatgtgaacgatgaggctgaagaagctgagttttatcaatatagtgggctagtttattacagattaaaccaataccttgtagctgcctctcatattgaacatgcaaaggttatctttgatcgtcttgaatatactgagccattattaaattgccaaattgtattagcaggtatttatcaagagttgcataaccctaaaaaagcagaggacatcttactagatgccttggaaaaggccacggataatgaggtaatgctggggttaattaatcgatccttagggctgaataaacttggcactaaagattacaaccaggcagaattttactttagacaagctttgtcttttaaggtgcacaaggatgcagcggttggagctaaaacaacatataatcttagtaatgttcttttcaaccaaggtaaccatgatgaggcaagaaagcagtttaaagcggctcatgcaggcgctaaatactacaaaaatcatgaatacatggcaagatgtctggcaacagaaggattgcatatcaaaaaagattacagtttagttgatacagcaatcgatgacctcaacaaattaggcttggactttgaagtggctgagg |
| *amyE* upstream | ttgatgggaatcacgagacaggttgcggttgaagcggttatgcttggagaaggggtcgtcaactgtgtgaacccgacatccggcgttctcatggcggtgcttgccgccagcggtattccgtatgtcaagtggctgcggtttatggtgccgcttgctctgatttggttcttgatcgggcttgtctttatcgtgatcggagtcatgatcaattgggggccgttttaacgattgctgcccgccggcttgtacggcgggcttttgagttattcattgcagaagcgcaggctgttattgtaacatgtaagccataagccattcgtaaaagtgcgggaggaaggtcatgaataatctgcgtaatagactttcaggcgtgaatgggaaaaataagagagtaaaagaaaaagaacaaaaaatctggtcggagattgggatgatagcgggagcatttgcgctgcttgatgtgatcatccgcggcattatgtttgaatttccgtttaaagaatgggctgcaagccttgtgtttttgttcatcattatcttatattactgcatcagggctgcggcatccggaatgctcatgccgagaatagacaccaaagaagaactgcaaaaacgggtgaagcagcagcgaatagaatcaattgcggtcgcctttgcggtagtggtgcttacgatgtacgacagggggattccccatacattcttcgcttggctgaaaatgattcttctttttatcgtctgcggcggcgttctgtttctgcttcggtatgtgattgtgaagctggcttacagaagagcggtaaaagaagaaataaaaaagaaatcatcttttttgtttggaaagcgagggaagcgttcacagtttcgggcagctttttttataggaacattgatttgtattcactctgccaagttgttttgatagagtgattgtgataattttaaatgtaagcgttaacaaaattctccagtcttcacatcggtttgaaaggaggaagcggaagaatgaagtaagagggatttttgactccgaagtaagtcttcaaaaaatcaaataaggagtgtcaagaatg |
| *amyE* downstream | tgagggcaaggctagacgggacttaccgaaagaaaccatcaatgatggtttcttttttgttcataaatcagacaaaacttttctcttgcaaaagtttgtgaagtgttgcacaatataaatgtgaaatacttcacaaacaaaaagacatcaaagagaaacataccctggaaggatgattaatgatgaacaaacatgtaaataaagtagctttaatcggagcgggttttgttggaagcagttatgcatttgcgttaattaaccaaggaatcacagatgagcttgtggtcattgatgtaaataaagaaaaagcaatgggcgatgtgatggatttaaaccacggaaaggcgtttgcgccacaaccggtcaaaacatcttacggaacatatgaagactgcaaggatgctgatattgtctgcatttgcgccggagcaaaccaaaaacctggtgagacacgccttgaattagtagaaaagaacttgaagattttcaaaggcatcgttagtgaagtcatggcgagcggatttgacggcattttcttagtcgcgacaaatccggttgatatcctgacttacgcaacatggaaattcagcggcctgccaaaagagcgggtgattggaagcggcacaacacttgattctgcgagattccgtttcatgctgagcgaatactttggcgcagcgcctcaaaacgtacacgcgcatattatcggagagcacggcgacacagagcttcctgtttggagccacgcgaatgtcggcggtgtgccggtcagtgaactcgttgagaaaaacgatgcgtacaaacaagaggagctggaccaaattgtagatgatgtgaaaaacgcagcttaccatatcattgagaaaaaaggcgcgacttattatggggttgcgatgagtcttgctcgcattacaaaagccattcttcataatgaaaacagcatattaactgtcagcacatatttggacgggcaatacggtgcagatgacgtgtacatcggtgtgccggctgtcgtgaatcgcggagggatcgcaggtatcactgagctgaacttaaatgagaaagaaaaagaacagttccttcacagcgccggcgtccttaaaaacattttaaaacctcattttgcagaacaaaaagtcaactaaccgcaactttagagtaaagggctgattgtcaatgtgggagcagttgtatgatccgtttggaaacgagtatgtgagcgcacttgtggcgctcactccgat |

**Table S3.** **Growth of S. clausii DSM 8716 in the presence of various antibiotics.** Sign (+) indicates visible growth, denoting resistance to the antibiotic; sign (–) indicates no growth, denoting sensitivity to the antibiotic. Highlighted boxes indicate antibiotic concentrations commonly used for selection in B. subtilis.

|  | 1 μg mL^-1^ | 5 μg mL^-1^ | 10 μg mL^-1^ | 20 μg mL^-1^ | 50 μg mL^-1^ | 100 μg mL^-1^ |
| --- | --- | --- | --- | --- | --- | --- |
| Ampicillin | **+** | **+** | **+** | **+** | **+** | **+** |
| Erythromycin | **+** | **+** | **+** | **+** | **+** | **+** |
| Nourseothricin | **-** | **-** | **-** | **-** | **-** | **-** |
| Spectinomycin | **+** | **+** | **+** | **+** | **-** | **-** |
| Kanamycin | **+** | **+** | **+** | **+** | **+** | **-** |
| Lincomycin | **+** | **+** | **+** | **+** | **+** | **+** |
| Tetracycline | **+** | **-** | **-** | **-** | **-** | **-** |
| Chloramphenicol | **+** | **+** | **+** | **-** | **-** | **-** |

**Figure S1.** Alignment of amino acid sequences of the xylose isomerase (XylA) from B. subtilis 168 and its homologue (XylA) in S. clausii DSM 8716. The multiple sequence alignment was generated using ClustalW as implemented in JALVIEW.


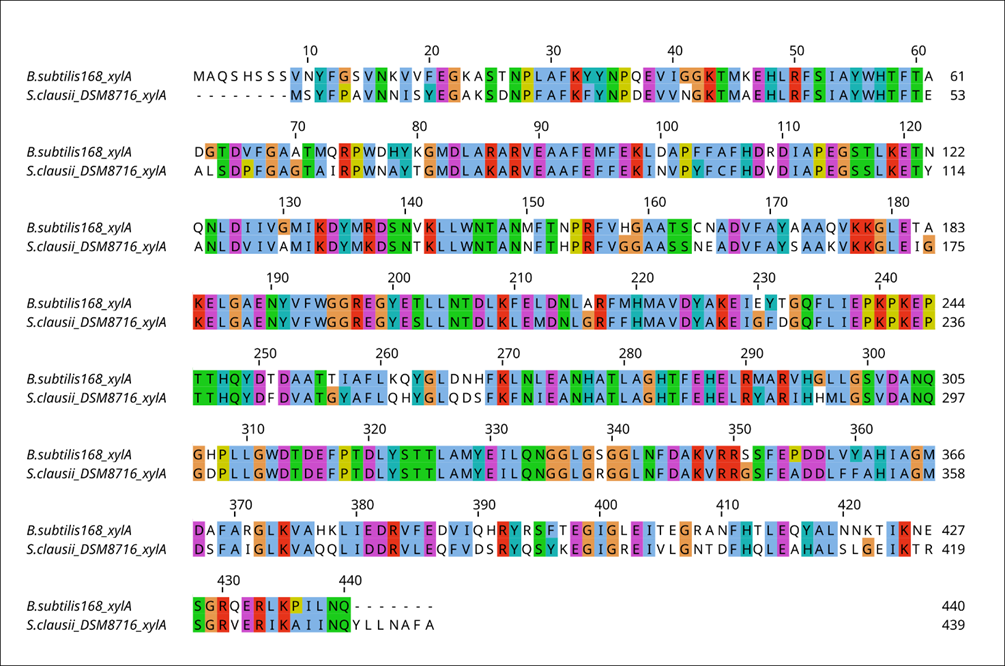


**Figure S2.** Alignment of amino acid sequences of the β-galactosidase GanA from B. subtilis 168 and its homologues (LacA) in S. clausii strains. The multiple sequence alignment was generated using ClustalW as implemented in JALVIEW.

**
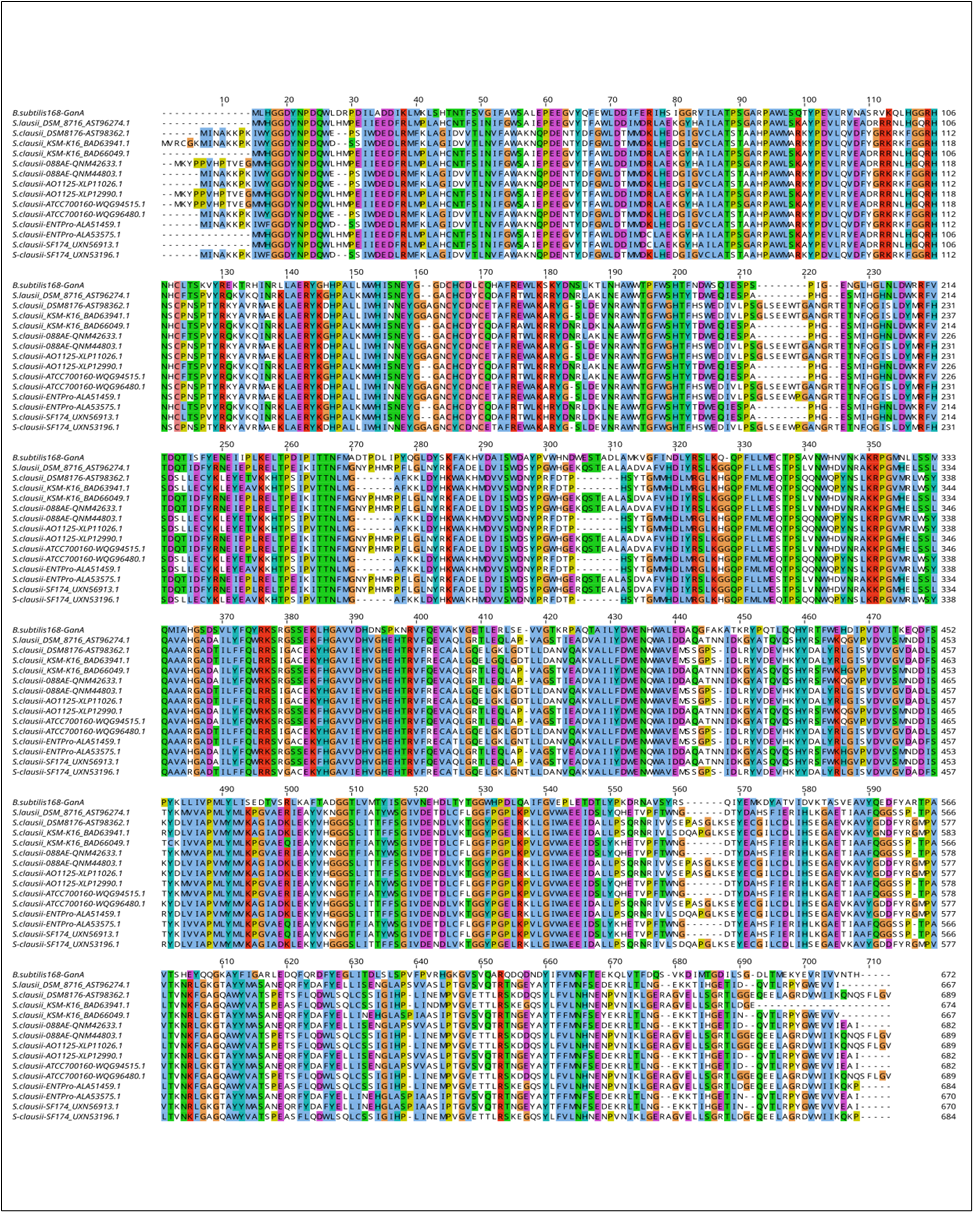
**

**Figure S3. Phylogenetic tree of B. subtilis β-galactosidases and various S. clausii strains.** The tree was constructed using sequences of the known GanA protein from B. subtilis 168 and the putative β-galactosidases from S. clausii, as described in the Materials and Methods section. Protein names are shown at the terminal nodes. The homologous LacA of S. clausii DSM 8716 is highlighted in red. The green box indicates the clade of putative LacA proteins.


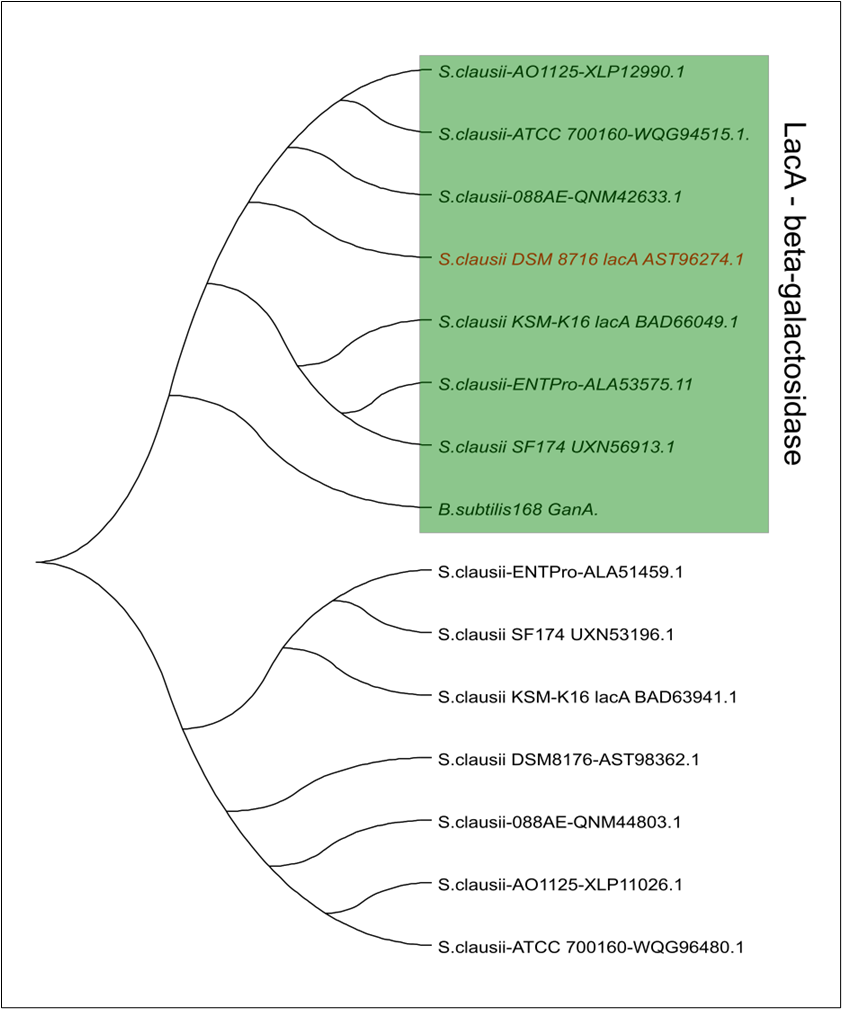

Supplement: Supplementary file 1 — Data S1: mbt270287‐sup‐0001‐DataS1.docx. [file MBT2-19-e70287-s002.docx]
